# Early Detection of Septic Shock Onset Using Interpretable Machine Learners

**DOI:** 10.3390/jcm10020301

**Published:** 2021-01-15

**Authors:** Debdipto Misra, Venkatesh Avula, Donna M. Wolk, Hosam A. Farag, Jiang Li, Yatin B. Mehta, Ranjeet Sandhu, Bipin Karunakaran, Shravan Kethireddy, Ramin Zand, Vida Abedi

**Affiliations:** 1 Steele Institute for Health Innovation, Geisinger Health System, Danville, PA 17822, USA; dmisra@geisinger.edu (D.M.); rs1444@scarletmail.rutgers.edu (R.S.); bkarunakaran@novanthealth.org (B.K.); 2 Department of Molecular and Functional Genomics, Geisinger Health System, Danville, PA 17822, USA; vavula1@geisinger.edu (V.A.); jli@geisinger.edu (J.L.); 3 Diagnostic Medicine Institute, Department of Laboratory Medicine, Geisinger Health System, Danville, PA 17822, USA; dmwolk@geisinger.edu (D.M.W.); hafarag@geisinger.edu (H.A.F.); 4 Critical Care Medicine, Geisinger Health System, Danville, PA 17822, USA; ybmehta@geisinger.edu (Y.B.M.); shravan.kethireddy@nghs.com (S.K.); 5 Neuroscience Institute, Geisinger Health System, Danville, PA 17822, USA; ramin.zand@gmail.com

**Keywords:** healthcare, artificial intelligence, machine learning, interpretable machine learning, explainable machine learning, septic shock, clinical decision support system, electronic health record

## Abstract

Background: Developing a decision support system based on advances in machine learning is one area for strategic innovation in healthcare. Predicting a patient’s progression to septic shock is an active field of translational research. The goal of this study was to develop a working model of a clinical decision support system for predicting septic shock in an acute care setting for up to 6 h from the time of admission in an integrated healthcare setting. Method: Clinical data from Electronic Health Record (EHR), at encounter level, were used to build a predictive model for progression from sepsis to septic shock up to 6 h from the time of admission; that is, *T = 1*, *3*, and *6 h* from admission. Eight different machine learning algorithms (Random Forest, XGBoost, C5.0, Decision Trees, Boosted Logistic Regression, Support Vector Machine, Logistic Regression, Regularized Logistic, and Bayes Generalized Linear Model) were used for model development. Two adaptive sampling strategies were used to address the class imbalance. Data from two sources (clinical and billing codes) were used to define the case definition (septic shock) using the Centers for Medicare & Medicaid Services (CMS) Sepsis criteria. The model assessment was performed using Area under Receiving Operator Characteristics (AUROC), sensitivity, and specificity. Model predictions for each feature window (1, 3 and 6 h from admission) were consolidated. Results: Retrospective data from April 2005 to September 2018 were extracted from the EHR, Insurance Claims, Billing, and Laboratory Systems to create a dataset for septic shock detection. The clinical criteria and billing information were used to label patients into two classes-septic shock patients and sepsis patients at three different time points from admission, creating two different case-control cohorts. Data from 45,425 unique in-patient visits were used to build 96 prediction models comparing clinical-based definition versus billing-based information as the gold standard. Of the 24 consolidated models (based on eight machine learning algorithms and three feature windows), four models reached an AUROC greater than 0.9. Overall, all the consolidated models reached an AUROC of at least 0.8820 or higher. Based on the AUROC of 0.9483, the best model was based on Random Forest, with a sensitivity of 83.9% and specificity of 88.1%. The sepsis detection window at 6 h outperformed the 1 and 3-h windows. The sepsis definition based on clinical variables had improved performance when compared to the sepsis definition based on only billing information. Conclusion: This study corroborated that machine learning models can be developed to predict septic shock using clinical and administrative data. However, the use of clinical information to define septic shock outperformed models developed based on only administrative data. Intelligent decision support tools can be developed and integrated into the EHR and improve clinical outcomes and facilitate the optimization of resources in real-time.

## 1. Introduction

Sepsis is a life-threatening condition that arises when the body’s response to an infection injures its tissues and organs as defined by the 1991 consensus [1,2,3]. Sepsis is a complex syndrome that is difficult to identify early, as its symptoms, such as fever and low blood pressure, overlap with those of other common illnesses. Without timely treatment, sepsis can progress to septic shock, which has a hospital mortality rate greater than 40%. Identification of sepsis patients who are at high risk of septic shock will be helpful for clinicians to prioritize preventive care and improve the survival rate. Early diagnosis, prompt antibiotic, and supportive therapy are associated with improved outcomes [4,5,6]. Severe sepsis and septic shock are the leading causes of morbidity and mortality in the Intensive Care Unit (ICU) [7]. Septic shock is a subset of sepsis with significantly increased mortality due to severe circulation and/or cellular metabolism abnormalities. During septic shock, the heart and circulatory system begin to fail and blood pressure drops. Septic shock, the leading cause of morbidity and mortality in the Intensive Care Unit (ICU), is costing the United States’ healthcare system more than $20 billion per year [8]. 

Translating recent advances in Artificial Intelligence (AI) to patient outcomes is an active area of research [9,10,11]. A few examples where AI has shown promise are interpreting chest radiographs [12], identifying malignancy in mammograms [10], and detecting incidental lung nodules analyzing computer tomography scans among others [13,14]. Leveraging data collected from the EHRs offers clinical insight, which can better augment favorable patient outcomes [15]. Data-driven AI models can also assign risk scores to transfer high-risk patients to intensive care units [16]. More and more advanced ML models are used to develop clinical decision systems, predicting in-hospital mortality, length of stay, readmission risk, and discharge diagnoses [17] and sepsis management [18,19]. In this study, we developed a working model for predicting septic shock in an acute care setting up to 6 h from the time of admission using real-time data. Predicting septic shock is challenging yet highly impactful, as timely diagnosis and prompt antibiotic and supportive therapy are associated with improved outcomes. This paper presents a practical working model for using ML to develop predictive models of septic shock in an Intensive Care Unit environment. The findings highlight how ML and large clinical and administrative data lakes can be leveraged to address practical challenges.

## 2. Related Works

Recent works have highlighted the unmet need for data-driven clinical decision systems for the identification of at-risk patients. For instance, in 2018, researchers [20] leveraged high-resolution time-series data to predict septic shock onset in the Intensive Care Unit, 4 to 12 h before the event. In 2019, it was demonstrated that [21] an expert AI system could outperform clinicians to predict sepsis onset. In 2020, Kim et al. [22] the possibility of predicting septic shock within 24 h using ML-based models was explored. Even though septic shock has higher mortality than sepsis [23], identification of both sepsis and septic shock patients in such a way to give the care providers more time (even a few hours) can lead to improved outcomes. Although there are many use cases of ML-based models of sepsis and septic shock, there is limited literature focusing on a working model in an integrated healthcare system focusing on scalability, real-time data access, and standardization of the sepsis and septic shock evolving phenotype definition. Previous works have focused on clinical models using various datasets and characteristics [24], focusing on the effect of ML algorithms on outcomes of sepsis patients.

**Figure 1:**
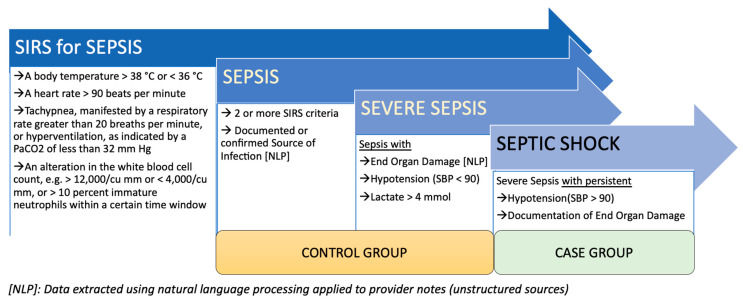
Case and control definition based on the SIRS criteria and Centers for Medicare & Medicaid Services (CMS) definition.

This project was part of an initiative to build a translational and interpretable decision support system as an assistive technology for our providers. In particular, we aimed to develop a prediction model of sepsis and severe sepsis to septic shock by using clinical data and comparing the model performance when only billing data are used to define the cohort. Data extraction from administrative sources (such as billing codes), which are in a structured form, is easier compared to data extraction from unstructured clinical sources (such as notes for extraction of the source of infection). The latter requires more complex queries, including the integration of natural language processing pipelines. It was [25] reported that identifying sepsis or septic shock patients based on clinical data, as compared to administrative data, is more accurate; however, many studies still rely mainly on administrative data. For septic shock, administrative data can be inaccurate as the patient’s progression to septic shock can occur at any time. While earlier works [26,27] have demonstrated moderate success using tree-based models for visit level prediction, recent works [26] leveraging temporal neural network-based models have shown promising results for predicting septic shock at visit and event levels. However, one of the challenges while defining cases and control revolves around the lack of consensus for defining sepsis and septic shock [7]. Cohort definition is the first and most important step of the modeling pipeline. In this study, we used clinical variables to map our cohort definition (cases: septic shock; controls: sepsis and severe sepsis [28]) with the Systemic Inflammatory Response Syndrome (SIRS) [29] criteria. The SIRS, as outlined by the Centers for Medicare & Medicaid Services [30], is outlined in Figure 1.

## 3. Methods

### 3.1. Data Sources

This study was approved by the Geisinger Institutional Review Board (IRB). Geisinger, an integrated multi-site health system in North Eastern Pennsylvania with a catchment population of approximately 2.5 million citizens, has been known for being one of the most “wired” and innovative healthcare systems in the United States. Thirteen years of retrospective data between April 2005 to September 2018 from EHR (EPIC), Insurance Claims and Billing (AMISYS), and Laboratory Systems (Sunquest) were used to create a sepsis dataset for this study. The systemic inflammatory response syndrome (SIRS) [30] criteria, outlined by Centers for Medicare & Medicaid Services (CMS) [31], were used to assign patients into the case and control groups—septic shock patients (case group) and sepsis and severe sepsis patients (control group). In production, the system was designed to detect septic shock using real-time data to assist clinicians when treating high-risk sepsis patients in ICU. In addition to the EHR data, billing codes were utilized to ascertain the correct diagnosis for a patient at a given encounter for comparative assessment.

The initial assessment of clinical features, which was based on input from the clinicians and the literature, resulted in 65 features in six different categories from the structured sources. The features included during the first assessment were broadly in the following categories: demographics, vitals, pathology and laboratory measurements, medications, comorbidities, and procedures. Additional variables, which are critical in sepsis and septic shock, were also considered. In particular, (1) use of vasopressors was part of the criteria to define septic shock (persistent hypotension), (2) use of antibiotic administration was also included in the study (to suspect infection), and (3) creatinine level was utilized to evaluate kidney function since the use of urine output data, also an important parameter, was challenging; the latter is associated with a high error rate, given the needs for visual assessment and manual data recording.

Data from structured and unstructured sources were extracted and processed. Clinical notes (unstructured sources) were used to ascertain clinical states, including the source of infection, focused exam, documentation of septic shock, and severe sepsis documentation. Medical ontology from the Unified Medical Language System (UMLS) [31] meta-thesaurus, including SNOMED [32], LOINC [33] and ICD-9/ICD-10 [34] were used in the data model abstraction. Technical details of the natural language processing (NLP) pipeline are provided in the data extraction section.

### 3.2. Feature Assessment

**Figure A1:**
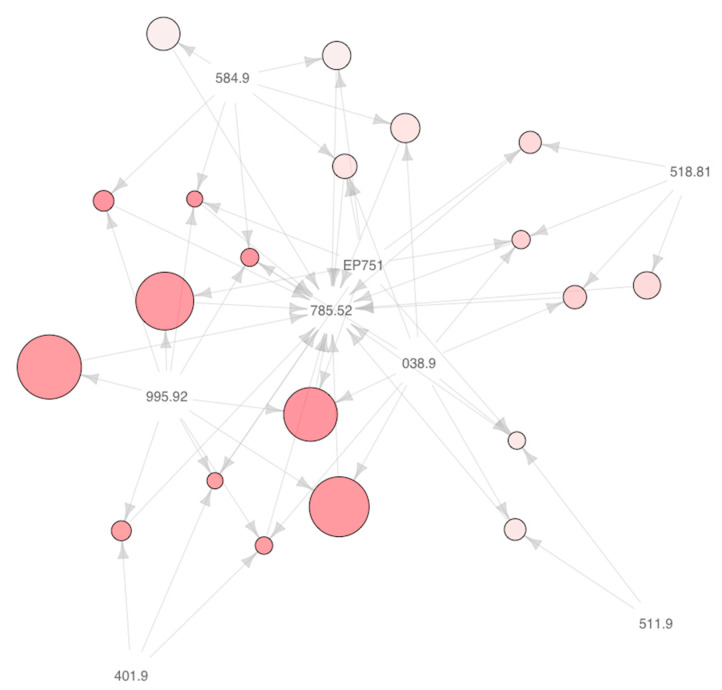
Graphical Representation of Association Rules with Septic Shock (ICD9 = 785.52) as a consequent. Diagnosis codes for the patients in the cohort were obtained and Association Rule Mining [35] was run to mine for relationships between comorbidities. In the study, “items” are diagnosis codes. Items are connected to rules using directional edges. For nodes representing rules, edges pointing from codes to rule vertices indicate antecedent items and an edge from a rule to an item indicates the consequent item. The reader is referred to [58] for more details about the visualization. 511.9: Unspecified pleural effusion; 401.9: Unspecified essential hypertension; 995.2: Severe sepsis; 038.9: Unspecified septicemia; 518.81: Acute respiratory failure; EP751: Other congenital anomalies of digestive system: 584.9: Acute kidney failure.

The list of features was further evaluated for the clinical implementation to ensure clear workflow integration. Stakeholders from the data management, EHR vendor (EPIC), Laboratory Medicine, and clinicians reviewed the comprehensive feature list, and a decision was made to include actionable features with high clinical value. The final list included the following features: blood culture, diastolic and systolic blood pressure, creatinine, lactic acid, mean arterial pressure (MAP), platelet count, pulse, respiration, temperature, white blood cell count, age, gender, height, and weight. Association Rule Mining [35] was also performed as part of the feature exploration strategy to investigate the relationship between comorbidities using diagnosis codes. Results from this additional assessment are included in the Appendix A (Figure A1) for the interested reader. 

### 3.3. Cohort Selection

Cohort definition involves establishing a reproducible process by which data elements from the EHR (both structured and unstructured) can be used to develop a longitudinal view of the patient. Deep phenotyping was performed to create different case and control cohorts based on structured and unstructured data sources. The Systemic Inflammatory Response Syndrome (SIRS) [30] criteria were used to group patients into the case (septic shock) and control (sepsis and severe sepsis) group (See Figure 1). Three different sets of case-control were also designed based on the adult patients (>18 years old) progressing from sepsis to septic shock at three different proceeding time frames from admission—*T = 1*, *3*, and *6 h* from the time of admission to septic shock progression (visit level early diagnosis—based on a left-align design). Since vitals are extracted directly from sensors and fed into the system as they are generated, our data was time-stamped, which allowed us to collect data points preceding the observation window. For instance, if there were three data points at 0.5 h, 2.5 h, and 3.5 h for a patient, for *T = 3 h* window, data at 2.5 h was utilized, similarly, for the *T = 6 h*, data point collected at 3.5 h was used and so forth.

### 3.4. Data Extraction

Analytics Infrastructure: Unified Data Architecture (UDA) is the Enterprise Data Lake providing core integration, storage, and user-specific access and retrieval information at Geisinger. It is an in-house 50-node cluster running with the capability to ingest, store, and transform big data using a combination of Apache Spark and Apache Hive on an Apache Hadoop cluster. Data from heterogeneous source systems and vendors (e.g., clinical, billing, radiology, laboratory) are ingested into an Enterprise Data Warehouse daily (EDW). The data model is used extensively for clinical reporting and advanced analytics. EDW was used as the source for the extraction of retrospective data and clinical features.

Data extraction from unstructured sources: Patient notes, specifically nursing notes, were used to determine the source of infection, chronic conditions, fluid bolus, and acute kidney disease. Apache cTAKES [36] was used as the natural language processing (NLP) engine. The NLP engine was modified to be utilized in a big data environment using the Apache Spark framework on Hadoop [37]. Concepts related to chronic conditions, fluid bolus, and acute kidney disease were identified from in-patient provider notes using entities from the UMLS meta-thesaurus. Notes with the relevant concepts were selected for downstream analysis. A custom regular expression-based NLP pipeline was applied to extract additional information for the three SIRS criteria, including the source of infection, chronic conditions, and fluid bolus. 

Data extraction from structured sources: Various data elements, including vitals, flowsheets, and medications were processed, enhanced, and integrated into Geisinger’s UDA platform. An Extract Transform Load (ETL) pipeline consolidated the data and aggregated clinical measures along with patients’ encounters and demographic information. This data was aggregated with unstructured patient notes to determine various events such as SIRS and Organ Dysfunction (OD). Sepsis, severe sepsis, and septic shock classification are performed based on these medical events’ chronology as defined by the CMS guidelines. The classified data was integrated with patients’ additional historical data such as chronic conditions and medical history. Finally, a longitudinal chronological narrative of various clinical measures and medical events from the time of admission was generated and used for model development. 

### 3.5. Data Processing

Various data processing, such as exploratory data analysis, imputation, and sampling, were performed before training and testing the various models. 

#### 3.5.1. Outlier Removal

The distribution of unique features was assessed to identify noise or outliers in the data. Units of the numeric variables and the bounds of lower and upper limits were applied (see Table A1). Furthermore, values identified outside of the six standard deviations were manually verified and removed if considered dubious.

#### 3.5.2. Imputation

Variables with more than 40% missing were excluded from the analysis. The only exception is lactic acid, which had an overall higher missingness; however, given the importance of this variable, a decision was made to include this key variable. The MICE package in R with the random forest implementation was used to impute missingness [38]. Given the large dataset, a custom pipeline was implemented using Apache Spark [39] and optimized for scalability. The distribution of variables before and after imputation was assessed to ensure consistency. 

#### 3.5.3. Class Imbalance

Given that the percentage of patients with septic shock (cases) is significantly smaller than patients with sepsis and severe sepsis (controls), three sampling strategies were applied. Statistical techniques were applied in the following specific order. First, Edited Nearest Neighbors (ENN) [40] was used to smooth the data distribution by removing misclassified samples based on nearest neighbors from the majority class. The ENN was followed by the Synthetic Minority Over Sampling Technique (SMOTE) [41] to increase the size of the minority class. Two different variations of SMOTE (SMOTE and Synthetic Minority Over-sampling Technique for Nominal and Continuous (SMOTE-NC)) were used for numeric and categorical features. Finally, under-sampling was addressed by using a random under-sampling (RUS) algorithm, applied to balance the classes [42]. Up-sampling, synthetically increasing the sample size of the minority class, was performed separately for labels from the Billing and CMS-based cohorts.

### 3.6. Modeling Strategy

Geisinger’s big data environment used for our modeling consisted of 34 physical nodes with 1140 vCores using 11.07 TB of memory. We also used the Yet Another Resource Negotiator (YARN) [37] cluster manager for jobs that are configured to use 200 executors with 5 GB memory container size. The technology stack used consisted of running spark jobs submitted to the YARN cluster resource manager.

As the list of features was limited to actionable features with the highest clinical utility, we did not perform data-driven feature selection; however, we used Pearson pairwise correlation analysis to corroborate that features in the cohort were not highly correlated. We split the data into training and testing (80/20 split) while retaining the proportion of classes. Model development was performed on 80% of the data, while model testing was performed on 20% of the data. During the model development (on the 80% of the data), 5-fold cross-validation was utilized. Furthermore, synthetic sampling was used only on the training data. Model performances were evaluated on the holdout test data set (20% of the data) using the area under the receiver operating characteristic curve (AUROC), specificity, and sensitivity. Consolidated metrics for 1, 3, and 6-h feature windows were also calculated. Thus, if the patient was assigned a septic shock label in any of the three time intervals, the consolidated prediction was selected as septic shock.

The models were derived from the two cohorts (cohort designed based on CMS criteria and billing information). Predicting the onset of septic shock in the proceeding *T* hours after admission was designed for *T = 1*, *3* and *6 h*. Time-dependent features (dynamic features) were collected for each window, and the results of the model performance were compared.

A total of eight different algorithms were trained: Logistic Regression [43], Regularized Logistic Regression [44], Bayes General Linear Model [45], Boosted Logistic Regression [46], C5.0 [46], Decision Trees [47], Support Vector Machine (SVM) [48], and Random Forest [49]. Grid search [50] was used to tune the hyperparameters for the classification models. Twenty node cluster, running Apache Spark, was used for tuning the models in conjunction with sparkR and R [39]. 

## 4. Results

### 4.1. Patient Characteristics

This study includes a total of 46,651 distinct adult patient (>18 years old) visits, extracted from Geisinger’s data warehouse between April 2005 and September 2018. Each record corresponds to a unique encounter. A set of 1226 records were excluded due to data quality and the excessive missing of static features such as height, gender, and age. The remaining 45,425 records met the inclusion criteria. 

Sepsis data sets for 1, 3, and 6 h feature windows had labels from CMS and Billing, depending on the data extraction process. There was a total of 3179 encounters from CMS (7% of the cohort) while billing-based septic shock records accounted for 6127 encounters—14% of the total records analyzed. Among the 45,425 records, 5784 were identified as a septic shock while 30,192 were identified as sepsis and severe sepsis (control) within a *T = 1 h* window; similarly, 5845 cases were classified as septic shock (cases) while 31,668 records were identified as controls within a window of *T = 3 h*. A total of 5852 records (cases) were septic shock while 32,329 records were sepsis (controls) within a *T = 6 h* window. Overall, 51% of all the cases and controls were men. The mean age was higher in the case group compared to the control group for the three case-control designs (*T = 1*, *3*, and *6 h* from admission). The same trend was observed for the average weight of the patients; however, the difference was marginal. Table 1 illustrates the cohort statistics for the *T = 1*, *3* and *6 h* prediction windows. This study was based on 15 features, including vitals, laboratory values, and baseline demographics. 

Our data showed that the average levels of lactic acid and creatinine were lower as the feature window is reduced to *T = 3 h *and *T = 1 h*. The average pulse followed the same trend (higher in the cases at *T = 6 h* versus *T = 1 h*). The average blood pressure had an opposite pattern; septic shock patients had on average lower blood pressure (both diastolic and systolic) at *T = 6 h*. The average temperature was lowest in the *T = 1 h* window for both case and control groups. Finally, the whole blood count (WBC) was lower in the case group compared to the control group for the three feature windows.

### 4.2. Machine Learning Models Can Be Trained for the Detection of Septic Shock Using Administrative Datasets

**Figure 2:**
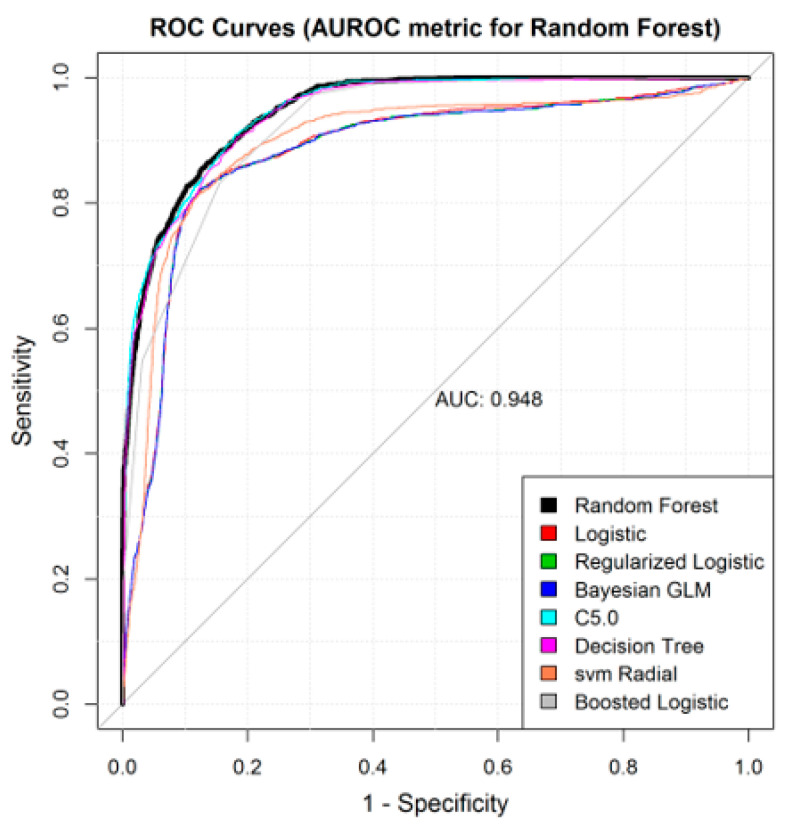
Receiver Operating Characteristic plots for the best machine learning algorithms.

**Figure A2:**
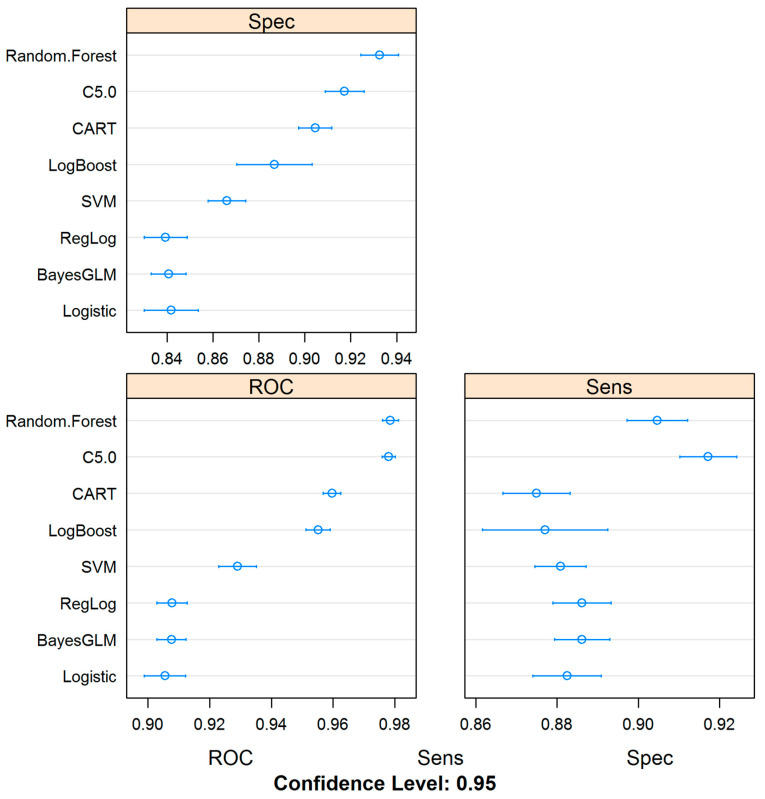
Comparison Performance Profiles based on aggregated data.

In this study, we used different case-control designs by focusing on different prediction windows, as well as labeling strategies—CMS versus billing information to label the cases. We also used a sampling technique to address the data imbalance. Overall, consolidated results demonstrated that clinical decision support systems can be developed for the detection of septic shock in ICU using administrative or clinical data. In the consolidated results, the final prediction label was determined based on whether at least one of the three case-control designs (based on the *T = 1*, *3*, or *6 h* windows) was able to detect septic shock (Table 2). Overall, four of the modeling algorithms resulted in an AUROC above 0.92, with an average AUROC of 0.91. The parameters for the grid search for the different models are also listed in Table 2. The average sensitivity and specificity of the consolidated results were 0.82 and 0.86 respectively. Finally, the best performance (AUROC of 0.943) was when Random Forest was used (Figure 2 and Table 2). The 95% confidence interval of the AUROC, sensitivity, and specificity are provided in Appendix A Figure A2. 

### 4.3. Model Prediction Performance Improves as the Time from Admission Widens

**Figure 3:**
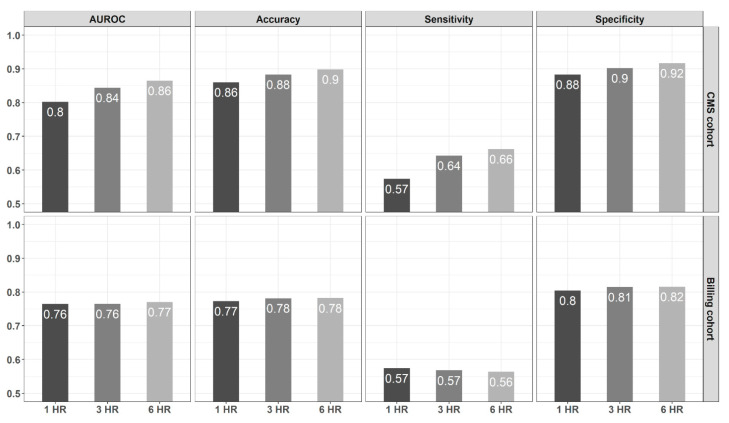
Consolidated Metrics, using Random Forest-based models, comparing CMS and Billing-based cohort as well as models based on the different windows, *T = 1*, *3*, and *6 h*.

Analysis of performance metrics, comparing the different case-control designs based on the feature window, demonstrated that the average model performance—in terms of AUROC, accuracy, sensitivity, and specificity—increased monotonically as time elapsed from admission increased from *T = 1 h* to *3* and *6 h* (Figure 3). Furthermore, our results on the best performing model using Random Forest also corroborated that the models based on the longer time frame (*T = 6 h*) consistently outperformed the others in terms of all performance metrics used in this study (Figure 3).

The prediction of models (at *T = 1*, *3*, *6 h*) are aggregated, such that the final prediction is true even if only one of the models labels that as true. This strategy reduced false negatives at the cost of false positives. Model AUROC, Specificity, Sensitivity, are reported in Table 2. It is important to indicate that the aggregate models for the best performing model are presented in Table 2 and the model performance metrics, especially model sensitivity and specificity, are above 0.8 for all the models.

### 4.4. Models Based on CMS-Derived Information Have Better Detection Power

Our results highlight that the prediction models when used in conjunction with labeling rules that are derived from CMS information (clinical information), rather than billing data (administrative information), can improve the performance metrics in terms of model AUROC, model accuracy, sensitivity, and specificity. Figure 3 shows that on average, model AUROC, sensitivity, specificity, and accuracy were higher for the CMS-based cohort for all three different case-control designs (*T = 1*, *3* and *6 h*). Model AUROC had the highest improvement for the 6 h window, with CMS-cohort reaching an average of 0.87, while billing-cohort for the same time frame reached an average of 0.77. Similarly, average model accuracy was highest for the same *T = 6 h* cohort when CMS information was used to define the cohort (0.90 versus 0.78 average accuracies). Model sensitivity and specificity were also higher with the CMS-based cohort (model sensitivity for *T = 6 h* is 0.66 versus 0.56; model specificity for *T = 6* is 0.92 versus 0.82). The same pattern was observed for the cohorts where the time from admission was defined as *T = 1* and *T = 3 h*.

### 4.5. Important Clinical Markers of Septic Shock

**Figure 4:**
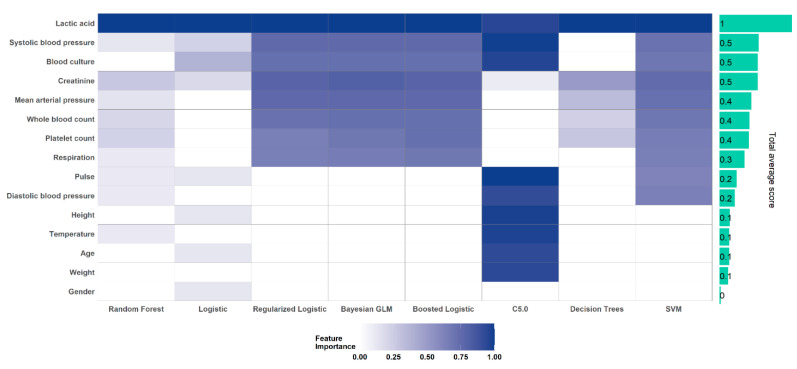
Feature Importance Profile for the eight machine learning models, based on aggregated measures.

Our results (Figure 4) demonstrated that the eight ML algorithms were able to identify lactic acid as the most important feature. Furthermore, there was a consensus in feature importance ranking in three out of the eight algorithms (logistic regression, regularized logistic regression, and Bayes generalized logistic regression). Overall, the dynamic features including laboratory features and vitals were important clinical markers for the majority of the algorithms. Demographic variables such as sex, age, and weight were the least discriminative variables by most of the models.

## 5. Discussion

This study demonstrated that machine learning models can be used to predict septic shock within the first 6 h of admission. Furthermore, model performance can be improved by aggregating the temporal models from each prediction window. Even when the rate of septic shock was between 7–14% (depending on how the septic shock is defined), the presented pipeline achieved a good balance of sensitivity and specificity at 1, 3, and 6 h from the time of admission. The major contribution of this study, is the use of a well-established framework, big data analytics and solid infrastructure in building interpretable decision support systems that can be integrated into clinical workflow in EHR.

### 5.1. Design Consideration for Building a Clinical Decision Support System for Detection of Septic Shock Using Healthcare Data

Our findings highlighted the value of data density for building predictive models. As the time from admission increases from *T = 1 h* to *3* and *6 h*, more clinical variables were available for each patient. The latter had an impact on model performance. This observation, even though expected, (a) can help design models with a balance between performance improvement versus how much time in advance a practitioner could be able to be notified of a patient’s declining condition, (b) corroborated the value of advances in laboratory technologies that can reduce the turn-around time, which could eventually facilitate the development of models that could target narrower windows as the data becomes available.

Our findings also demonstrated that the cohort definition for a clinical application can benefit if clinical information is leveraged as opposed to relying only on administrative (billing) information. The latter might be counter-intuitive, as billing codes may be more robust, at least for some conditions. Administrative data tend to be considered in many studies as a gold standard since billing codes are entered after chart review and have legal implications. However, as our results corroborated, clinically derived criteria using data from structured and unstructured sources, such as SIRS criteria, can exhibit higher fidelity in identifying septic shock patients when compared to leveraging only diagnostic codes. 

Besides a carefully-designed cohort definition and selection of the optimal prediction window (based on clinical workflow settings and turn-around time to have patient-level data for the model), we discussed important technical considerations for building a successful ML-enabled decision support system. One such consideration was to address the class imbalance between cases and controls. Our results denoted the value of applying robust sampling strategies to address the challenges due to the imbalanced nature of the dataset. Even though we did not compare our model performance with and without sampling as a pre-processing step, evidence suggests that this design strategy likely aided our model performance. Fleuren et al. [51], in their systematic review of ML-based models of sepsis, identified that some of the studies [51] potentially suffered from selection bias. In particular, to label septic shock patients, authors [5] used discharge ICD9 codes for acute infection to identify acute organ dysfunction and complemented that information with the need for vasopressors within 24 h of ICU transfer. In another study [27], authors used deep learning models to assess risk score 4 h before onset. In essence, since many patients present themselves in the Emergency Department with imminent or overt symptoms of septic shock, it is important that a decision support system, when integrated into the clinical workflow, can detect septic shock patients; therefore, in our design strategy, we ensured patients with imminent or overt septic shock were included to mimic a realistic situation. Finally, as EHR provides a valuable resource, it is important to leverage scalable analytical frameworks (such as pre-processing, data augmentation, use of ontologies, etc.) for providing assistive tools to providers in real-time. 

### 5.2. Lactic Acid and Other Laboratory Measurements are Highly Important Indicators of Progression to Septic Shock

Epidemiological studies have established that the initial hypotension and lactic acid levels are important indicators of the progression of sepsis to septic shock [52,53]. Our results also highlighted that lactic acid is the most important indicator of septic shock followed by blood culture, creatinine level, and systolic blood pressure. However, it should be mentioned that lactic acid demonstrated higher than 40% overall sparsity, yet, it was decided to include this important variable in the model. In our dataset, lactic acid was not missing completely at random, as the missing level in the control group was higher; our data included 25,352 encounters out of 43,332 with lactic acid data available in the control group, versus 6037 encounters out of 6486 with lactic acid in the case group, for the 6-h window. Our team is leading comprehensive studies in the imputation of laboratory values [54] and we hope in the follow-up study we can better address this challenge. 

Overall, other laboratory values were found to be relevant to the decision support system. Early warning scores do not consider laboratory values, however, in a recent meta-analysis of 28 studies [53] it was observed that overall laboratory values play an important role. Static features (age, sex, height, and weight) are the least important variables in the majority of the models used in this study. Furthermore, as different algorithms demonstrated different patterns (see Figure 4), it is imperative to not only rely on one modeling algorithm but an ensemble of models [55] when building prediction models based on a limited set of variables for time-critical conditions. 

### 5.3. Strengths, Limitations, and Future Work

This study had several strengths and some limitations. Using a large dataset from an integrated healthcare system was a clear strength; however, Geisinger’s patients’ cohort were predominantly Caucasians, therefore, models developed in this study may not be generalizable to other healthcare systems without further fine-tuning and optimization. Furthermore, the use of large clinical data leads also to a study limitation. Data from the EHR tend to be noisy; however, with the proper data extraction pipeline and close collaboration with the clinical team, it is possible to augment data quality and reduce systemic bias. However, models developed using EHR-based data can be integrated and deployed into the same healthcare system more effectively, as ML models trained on the data specific to a particular healthcare system (and population) can provide better specificity and sensitivity. 

Another strength and key contribution of this study is the development and comparison of two cohorts, based on administrative and clinical data, using billing information and clinical information based on CMS guidelines. Other studies have relied on using clinical markers such as lactic acid levels in combination with hypotension for determining septic shock [53]; however, the progression from sepsis to septic shock occurs on a spectrum and there are specific criteria that define this progression, from sepsis to severe sepsis and eventually to septic shock, the latter is clearly defined by the CMS guidelines [53,56]. Our results showed that the clinically derived cohort is more robust and leveraging guideline recommendations can improve the performance of the models. However, since the use of SIRS criteria may also lead to labeling bias (over-diagnosed cases), it is important to work closely with the clinical team and consider additional guidelines and metrics as needed. It is also important to perform a careful evaluation and comparative analysis (such as targeted chart review, etc.). Also, given the study limitation around the use of SIRS criteria, the strategy in this study was to align our decision support system with the contemporaneous roll-out of the CMS sepsis protocol, which did not include qSOFA or SOFA at the time this study was conceptualized. As in any healthcare system, with changing recommendations and guidelines, we are working on adapting our models with clinical workflow accordingly. Finally, since we use a multi-level approach in defining our cohort, our strategy is robust and can be updated relatively efficiently based on new guidelines. In particular, we use ICD codes as the first level, complemented with clinical data from notes and other sources of structured data. It is important to emphasize that diagnosis codes may have a systemic bias as they are intended for billing purposes. Furthermore, our case/control ratio had a significant imbalance, which typically leads to a reduction in model performance. However, as the field of machine learning is advancing at an unprecedented rate, we are exploring the use of novel strategies (such as the use of the generative adversarial network (GAN)) [57], which could be used to address data imbalance and to improve our models.

As future directions, our team is actively working on further refining our septic early detection models based on technical and clinical advances. In particular, (1) some of the important data elements such as SOFA score (and different variations of SOFA score) were not captured in our EHR routinely at the time of this study. Given the clinical utility of such data elements, our system is now capturing these important variables more consistently. Therefore, as part of future work, we will be integrating these new variables and assessing their predictive utility. (2) Certain variables, especially laboratory variables (such as blood cultures), have a higher turn-around time (sometimes ranging between 48 to 72 h). In this study, we used the presence of blood culture order as a binary variable; however, having the actual test results could improve the detection of septic shock. We are working on integrating more laboratory-based features as their turn-around time improves. Finally, (3) many other laboratory variables could be included in the model; however, laboratory values tend to suffer from non-at-random missing and at high rates, and imputing them is a challenging task. Our team is developing imputation modeling that is designed specifically for laboratory-based features [54]. We believe better imputation and more targeted hyperparameter tuning, including sensitivity-based analysis, could further improve model performance.

One of the main limitations of this study design is that some patients who progress to septic shock might be mislabeled as controls in the cohort. Even though this can be avoided by taking a large time window and leveraging pathology results, the technical and clinical steps needed to address this study limitation are manifold and beyond the scope of this study. Currently, the turnaround time for pathology reports makes it impractical for the integration of such data into a decision support system that is aimed at assisting ICU providers in real-time-few hours after the patient is admitted to ICU. Another potential source of noise is the intervention by care providers e.g., administration of fluid bolus based on capillary refill, which would suppress clinical markers e.g., SBP, lactate to baseline, thus misleading the model during training.

Furthermore, it is difficult to know the impact of antibiotics on the specific trajectory of an individual patient as infection types are different and outcomes of progression are predicated based on many dynamic variables. For instance, it has been shown that 30% of patients who received appropriate anti-infective before the onset of hypotension continued to develop septic shock [12]. Thus, more targeted research is needed to assess the impact of medication at a personalized level before such information can be used for practical and time-sensitive applications. 

Finally, this study is unique as it operates directly on the multiple sources of clinical data to build an ML-based decision support system for the detection of septic shock. This study also demonstrated that high-resolution and large heterogeneous data sets from administrative sources can be used to develop assistive tools for time-sensitive conditions such as the progression of sepsis or severe sepsis to septic shock. Such technologies could be integrated into the electronic healthcare system to improve the detection of septic shock and enable optimization of resources. The models have the potential to improve clinical outcomes in real-time.

## Figures and Tables

**Table 1:** Cohort Statistics based on CMS criteria.

SEPSIS DATASET	1 H		3 H		6 H	
	Cases	Controls	Cases	Controls	Cases	Controls
PATIENTS, N	5784	30,192	5845	31,668	5852	32,329
ENCOUNTERS, N	6409	40,242	6475	42,475	6486	43,332
MALE, N(%)	3322(51)	18,468(51)	3355(51)	19,130(51)	3360(51)	17,984(49)
MEAN AGE(SD)	51(27)	48(29)	65(19)	62(21)	65(19)	62(21)
MEDIAN AGE(IQR)	56(11–101)	50(5–95)	67(44–90)	67(42–92)	69(46–92)	66(41–91)
MEAN WEIGHT(SD)	166.55(76.46)	158.13(81.50)	179.34(67.18)	178.75(71.28)	179.30(67.26)	178.51(71.51)
**VITALS, MEAN(SD)**						
DIASTOLIC BP	72.3(16.6)	73.8(16.9)	63.2(20.8)	67.4(17.9)	63.2(20.8)	67.3(17.9)
SYSTOLIC BP	129.8(26.3)	129.2(25.6)	111.0(29.4)	123.5(28.1)	110.9(29.5)	123.3(28.2)
PULSE	95.80(27.06)	101.54(28.30)	108.20(26.23)	100.89(24.65)	108.22(26.26)	100.83(24.69)
RESPIRATION	20.90(8.04)	21.92(9.08)	23.46(8.53)	21.64(7.85)	23.49(8.64)	21.65(7.93)
TEMPERATURE	98.59(1.91)	98.84(1.99)	99.32(2.94)	99.44(2.33)	99.29(2.93)	99.41(2.32)
MAP ^1^	92.14(18.02)	92.55(17.73)	79.91(22.15)	86.59(19.20)	79.66(22.30)	85.96(19.58)
GCS ^2^	4.93(0.40)	4.95(0.32)	4.76(0.76)	4.88(0.51)	4.75(0.77)	4.88(0.51)
**LABORATORY MEASURES, MEAN(SD)**						
CREATININE	1.446(1.445)	1.459(1.470)	1.912(1.637)	1.645(1.605)	1.914(1.650)	1.645(1.610)
LACTIC ACID	2.59(2.49)	2.07(1.38)	4.48(3.53)	2.15(1.50)	4.51(3.54)	2.12(1.46)
APTT ^3^	35.17(12.56)	35.17(11.57)	37.24(13.86)	36.49(12.38)	37.45(14.09)	36.56(12.43)
PLATELET COUNT	231.20(101.84)	237.76(106.06)	221.66(126.62)	231.20(120.81)	220.82(126.11)	231.10(121.14)
PT/INR ^4^	1.55(0.94)	1.53(0.90)	1.74(1.09)	1.61(0.95)	1.77(1.12)	1.61(0.96)
WBC	15.33(10.82)	13.98(9.34)	15.47(11.12)	13.99(9.93)	15.47(11.12)	13.95(9.93)

^1^ Mean Arterial Pressure; ^2^ Glasgow Coma Score; ^3^ Activated Partial Thromboplastin Time; ^4^ Prothrombin Time Test.

**Table 2:** Performance metrics for the best model for each machine learning algorithm.

MODELS	AUROC	SENSITIVITY	SPECIFICITY	HYPER PARAMETERS	TUNED HP VALUES
**RF**	0.9483	0.8392	0.8814	mtry, maxTree, maxdepth	2, 1000, 4
**C5.0**	0.9474	0.8087	0.8944	Model, Winnowing, Boosting Iterations	Rules, False, 20
**DT**	0.9436	0.8553	0.8577	Complexity Parameter	0.000351617
**BL**	0.9239	0.8328	0.8448	Boosting Iterations	31
**SVM**	0.8962	0.8336	0.851	Sigma, Cost	0.01898621, 16
**LR**	0.8839	0.8304	0.8622		
**RLR**	0.8821	0.8288	0.8615	Cost, Loss Function, Epsilon	2, L1, 0.001
**BGLM**	0.882	0.828	0.8625		

RF: Random Forest, DT: Decision Trees, BL: Boosted Logistic, SVM: Support Vector Machine (Radial), LR: Logistic Regression, RLR: Regularized Logistic Regression, BGLM: Bayes Generalized Logistic Regression, HP: hyper-parameters.

## Data Availability

The data analyzed in this study is not publicly available due to privacy and security concerns. A GitHub link to the team’s notebook with exploratory data analysis, additional meta-data and summary plots are compiled for reference: https://github.com/TheDecodeLab/early_sepsis_detection_2020.

## Appendix A

**Table A1:** Upper and lower limits on variables as part of the data pre-processing for variables that had outliers after considering six standard deviations.

*MEASURE*	*LOWER_LIMIT*	*UPPER_LIMIT*
Temperature	96.8	101
Heart rate (pulse)		90
Respiration		20
White blood cell count	4000	12,000
Systolic blood pressure (SBP)	90	
Mean arterial pressure	65	
SBP decrease	Baseline-40	
Creatinine		2
Urine output	0.5	
Bilirubin		2
Platelets	100,000	
INR ^1^		1.5
APTT ^2^		60
Lactate		2

^1^ Prothrombin Time and International Normalized Ratio; ^2^ Activated Partial Thromboplastin Time.
